# Supplementary material for: Polyhydroxyalkanoate/Antifungal Polyene Formulations with Monomeric Hydroxyalkanoic Acids for Improved Antifungal Efficiency
Source: Antibiotics (Basel). 2021 Jun 18;10(6):737. doi: 10.3390/antibiotics10060737 (PMC8234488; doi:10.3390/antibiotics10060737)
Supplement: Supplementary file 1 [file antibiotics-10-00737-s001.zip › antibiotics-1253331-supplementary.pdf]

Supporting Information

Polyhydroxyalkanoate/antifungal polyene formulations with monomeric hydroxyalkanoic acids for improved antifungal efficiency

Marina Pekmezovic [1,2], Melina Kalagasidis Krusic [3], Ivana Malagurski [1], Jelena Milovanovic[1], Karolina Stępień [4], Maciej Guzik [5], Romina Charifou [6], Ramesh Babu [6], Kevin O'Connor [7] and Jasmina Nikodinovic-Runic [1,*]

[1] Institute of Molecular Genetics and Genetic Engineering, University of Belgrade, Vojvode Stepe 444a, 11221 Belgrade, Serbia; marinapekmezovic@gmail.com
[2] Department of Microbial Pathogenicity Mechanisms, Leibniz Institute for Natural Product Research and Infection Biology, Hans Knoell Institute, Beutenberstrasse 11a, 07745, Jena, Germany
[3] Faculty of Technology and Metallurgy, University of Belgrade, Karnegijeva 4, 11000 Belgrade, Serbia; meli@tmf.bg.ac.rs
[4] Department of Pharmaceutical Microbiology, Centre for Preclinical Research and Technology, Faculty of Pharmacy, Medical University of Warsaw, Banacha 1B, 02-097 Warsaw, Poland; kstepien@wum.edu.pl
[5] Jerzy Haber Institute of Catalysis and Surface Chemistry Polish Academy of Sciences, Niezapominajek 8, 30-239 Krakow, Poland; maciej.guzik@ikifp.edu.pl
[6] AMBER Centre, CRANN Institute, School of Chemistry, Trinity College Dublin, Dublin, Ireland; babup@tcd.ie
[7] BiOrbic Bioeconomy SFI Research Centre and School of Biomolecular and Biomedical Sciences and Centre for Synthesis and Chemical Biology, University College Dublin, Belfield, Dublin 4, Ireland; kevin.oconnor@ucd.ie

* Correspondence: jasmina.nikodinovic@gmail.com; jasmina.nikodinovic@imgge.bg.ac.rs Tel.: +381 11 397 60 34

Table S1. Values of melting temperatures and enthalpies obtained by DSC (1st heating) and values of the maximum decomposition temperatures determined by TGA

	**Control**	**NYS-9**	**NYS-17**	**AMB**	**AMBM**
**T_m (°C)**	49.31	49.22	49.77	44.19	44.28
**ΔH_m (J/g)**	25.67	21.59	24.09	22.63	21.62
**T_{max} (°C)**	256.6	268.0	258.1	264.7	260.0

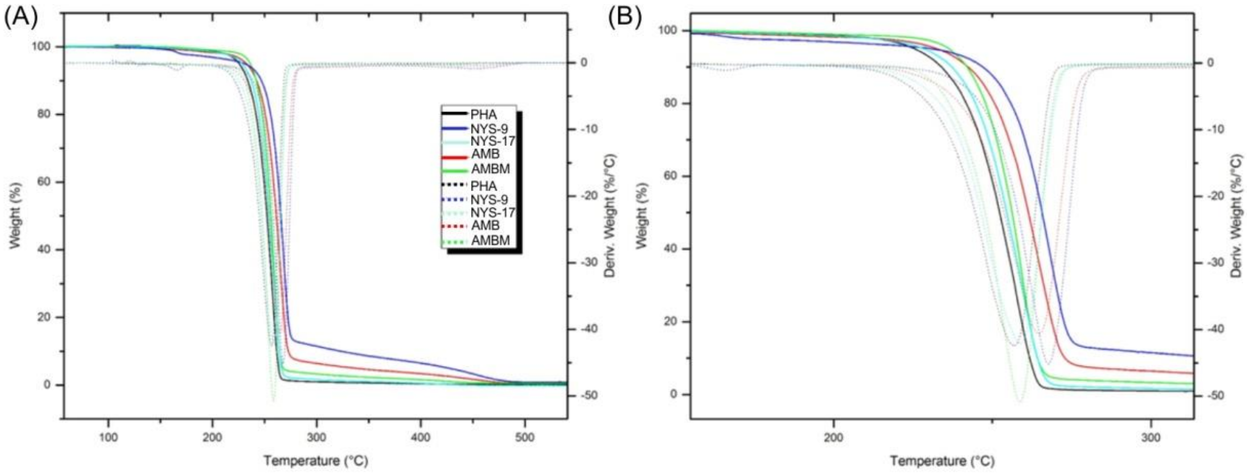

Figure S1. TGA thermograms obtained for the different samples (A) and close up (100 °C – 300 °C) of the TGA thermograms (B).

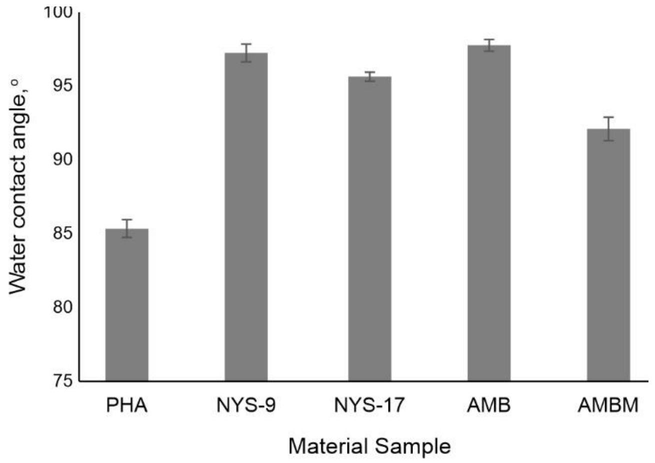

Figure S2. Water contact angle of PHA materials. The values are represented as mean ± standard deviation.

Table S2. Zones of inhibition [mm] of PHA films containing polyenes against clinical *Candida* spp. strain.

	***Candida albicans* 329UCK**
**Material**	Inhibition zone [mm]
**NYS-17**	26
**NYS-9**	22
**AMBM**	19
**AMB**	14
**PHA**	0
